# Dynamics of serological responses to defined recombinant proteins during *Schistosoma mansoni* infection in mice before and after the treatment with praziquantel

**DOI:** 10.1371/journal.pntd.0008518

**Published:** 2020-09-11

**Authors:** Eman Sayed Mohammed, Risa Nakamura, Yombo DJ Kalenda, Sharmina Deloer, Taeko Moriyasu, Mio Tanaka, Yoshito Fujii, Satoshi Kaneko, Kenji Hirayama, Ahmed I. Ibrahim, Mahmoud A. El-Seify, Asmaa M. Metwally, Shinjiro Hamano

**Affiliations:** 1 Department of Parasitology, Faculty of Veterinary Medicine, South Valley University, Qena, Egypt; 2 Department of Parasitology, Institute of Tropical Medicine (NEKKEN), Nagasaki University, Nagasaki, Japan; 3 Program for Nurturing Global Leaders in Tropical and Emerging Communicable Diseases, Graduate School of Biomedical Sciences, Nagasaki University, Nagasaki, Japan; 4 Department of Eco-epidemiology, Institute of Tropical Medicine (NEKKEN), Nagasaki University, Nagasaki, Japan; 5 Nagasaki University Nairobi Research Station, NUITM-KEMRI Project, Nairobi, Kenya; 6 Department of Immunogenetics, Institute of Tropical Medicine (NEKKEN), Nagasaki University, Nagasaki, Japan; 7 Department of Poultry Disease, Faculty of Veterinary Medicine, South Valley University, Qena, Egypt; 8 Department of Parasitology, Faculty of Veterinary Medicine, Kafrelsheikh University, Egypt; The University of Melbourne, AUSTRALIA

## Abstract

To eliminate schistosomiasis, appropriate diagnostic tests are required to monitor its prevalence and transmission, especially in the settings with low endemicity resulting from the consecutive mass drug administration. Antibodies that react with either crude soluble schistosome egg antigens or soluble worm antigen preparations have been used to monitor infection in low-prevalence regions. However, these detection methods cannot discriminate current and past infections and are cross-reactive with other parasites because both antigens contain numerous proteins and glycans from schistosomes, and standard preparations need maintenance of the life cycle of the schistosome. To evaluate the potential utility of nine recombinant *Schistosoma mansoni* proteins as single defined antigens for serological diagnosis, we monitored the kinetics of antibodies to each antigen during *S*. *mansoni* infection in mice before and after the treatment with praziquantel. C57BL/6 mice were infected with 50 cercariae. The levels of immunoglobulin G (IgG) raised against five recombinant antigens (RP26, sm31, sm32, GST, and LAP1) significantly increased as early as 2–4 weeks after infection and rapidly declined by 2 weeks after the treatment, whereas those raised against crude *S*. *mansoni* egg antigens or other antigens remained elevated long after the treatment. The IgG1 raised against RP26, sm31, and serpin decreased after the treatment with praziquantel, whereas the IgE raised against serpin declined strikingly after the treatment. This study clarifies the dynamics of the serological responses to recombinant *S*. *mansoni* proteins during infection and after the treatment with praziquantel and identifies several candidate antigens with potential utility in the monitoring and surveillance of schistosomiasis toward the elimination of schistosomiasis.

## Author summary

Schistosomiasis is the second most important human parasitic disease in tropical and subtropical areas: estimates show that at least 218 million people required treatment in 2015 and remains a major Neglected Tropical Disease impacting the health of the poorest populations. The global strategy for schistosomiasis control is focused on eliminating disease through periodic, large-scale population treatment with praziquantel (PZQ). With the progress towards the control and the elimination of schistosomiasis by mass drug administration (MDA) with PZQ, more sensitive diagnostics that can monitor the dynamics of schistosomiasis transmission are required to instruct MDA programs and assess reinfection. Detecting antibodies react with either crude soluble schistosome egg antigens or soluble worm antigen preparations have been used to monitor infection in low-prevalence settings. However, these detection methods cannot discriminate current and past infections and are cross-reactive with other parasites because it contains numerous proteins and glycans from schistosomes, and standard preparations need maintenance of the life cycle of the schistosome. This study clarified that the dynamics of the serological responses to defined recombinant proteins during *S*. *mansoni* infection in mice before and after the treatment with PZQ and identifies several candidate antigens with potential utility in the monitoring and surveillance of schistosomiasis toward the elimination of schistosomiasis.

## Introduction

Schistosomiasis, a chronic helminthic disease, is the second most devastating tropical parasitic disease after malaria. It is a major cause of morbidity and mortality and remains a public health concern affecting the poorest populations in developing countries [1]. Epidemiological studies have estimated that more than 218 million people throughout the world are infected with schistosomes and more than 250,000 deaths per year are related to the disease and its subsequent complications [2]. *Schistosoma mansoni* is one of the major species of the genus *Schistosoma* that infect humans and is widely spread in sub-Saharan Africa, some parts of the Middle East, Central and South Americas, and some West Indian islands [3, 4]. The pathogenesis of the disease is associated with the location of the adult worms in the blood vessels of the infected host, where adult females release eggs during their long lifespan. These eggs are trapped in host tissues, such as the liver, intestine, and pelvic venous plexus, causing inflammation and pathogenicity [4, 5]. The host immune responses to these eggs lead to the formation of granulomas around the eggs and collagen deposition, with consequent liver damage [6].

The current strategy to control and eliminate schistosomiasis relies on mass drug administration (MDA) using praziquantel (PZQ) in endemic areas. However, to guide control programs and to facilitate post-elimination surveillance, novel sensitive, affordable, and user-friendly diagnostics are urgently required. The envisioned test(s) should be able to discriminate between current and previous schistosome infections in low-intensity settings and/or to detect very light infections in near-elimination settings. The Kato–Katz (KK) thick smear method is the traditional method for detecting helminthic eggs, including schistosome, in stools [7–9]. This method is simple and can differentiate *Schistosoma* species with high specificity, but lacks sensitivity in geographic regions with low disease prevalence and requires trained technicians, microscopy, and electricity [10]. The KK test also does not detect premature infection because it takes 4–6 weeks for schistosomula to develop into adults and start laying eggs [11–13]. To overcome the limitations of this method, the simultaneous application of different diagnostic tests has been used to monitor the transmission status of schistosomiasis. Serological methods that detect antibodies against crude antigens prepared from schistosomes have been included to improve the sensitivity of screening possibly infected individuals and to monitor the transmission status of the organism. Soluble egg antigens (SEA) and soluble worm antigen preparations (SWAP) have been studied and used for this purpose [14]. Because both SEA and SWAP are crude antigens containing numerous proteins and glycans derived from schistosomes, the detection method has the disadvantages of cross-reactivity with other parasites, its inability to discriminate between current and past infections, and the difficulty of formulating the standard preparations because the whole life cycle of schistosomes must be maintained using both its final hosts and intermediate host snails [15]. Because the eggs remain in the host after the treatment with PZQ and continue to stimulate the host’s immune responses to the egg-derived antigens, antibodies directed against the crude antigens are maintained at high levels long after the treatment, and 1–2 years may be required for them to decline to the basal levels after the treatment [16]. Currently, a point-of-care test to detect urinary circulating cathodic antigen (POC-CCA) is available for monitoring *S*. *mansoni* infections [17]. This method has many advantages, including its ability to discriminate current and past infections because it detects circulating antigens in the urine produced by the living adult worm, whereas a serological test is expected to show superior sensitivity in low-intensity and post-elimination contexts [18, 19].

Therefore, there is a critical need for novel sensitive diagnostics that can guide schistosomiasis control programs and facilitate its post-elimination surveillance. The detection of antibodies against recombinant antigens may provide an alternative method that can discriminate between current and previous schistosome infections in low-intensity settings and detect very light infections in near-elimination settings. Antibody levels have been used to detect schistosome infection and to assess the elimination of these infections in some countries [20]. The limitations of this approach are attributable to the persistence of these antibodies after cure, so identifying particular candidate antigens that induce sharp short-lived antibody responses would overcome this limitation [20]. From the various antigens of *S*. *mansoni*, a panel of nine previously reported antigens was selected to examine the dynamics of the serological responses to recombinant *S*. *mansoni* proteins [21]: RP26 (sm22.3, LGG) [22], cathepsin B (sm31) [23, 24], hemoglobinase (sm32) [24], serine protease inhibitor (serpin) [25], filamin [26–28], tropomyosin [29], glutathione S-transferase (GST) [30, 31], leucine aminopeptidase 1 (LAP1), and leucine aminopeptidase 2 (LAP2) [32]. The dynamics of immunoglobulin G4 (IgG4) and IgE raised against these recombinant antigens may provide useful information. In this study, we tested the potential utility of these *S*. *mansoni* antigens, by assessing the kinetics of specific Ig levels in sera, to monitor the status of *S*. *mansoni* infection after the treatment with praziquantel (PZQ).

## Materials and methods

### Ethics statement

The study protocol was approved by the Committee for Ethics on Animal Experiments (approval number 1505181226) in Nagasaki University. All of the studies were conducted under the guidelines for animal experiments, Nagasaki University and according to Japanese law for Humane Treatment and Management of Animals (Law No. 105 dated 19 October 1973 modified on 2 June 2006).

### Parasite

A Puerto Rican strain of *S*. *mansoni* was used in this study. The life cycle of this parasite is maintained at the Animal Facility of Nagasaki University, with the successive passage of the parasite through *Biomphalaria glabrata* snails and ICR mice or jirds. ICR mice were percutaneously infected with 250 cercariae and killed 7 weeks after infection, and the adult worms were collected from their portal veins with intracardiac phosphate-buffered saline (PBS). Eggs were isolated with PBS from the livers of the infected mice, as previously described [33].

### Animals, infection, and treatment

Seven-week-old female C57BL/6 mice were purchased from SLC (Shizuoka, Japan) and housed at the Animal Facility of Nagasaki University. The mice were kept under environmentally controlled, specific-pathogen-free conditions, with free access to food and water. Animal housing, handling, and feeding complied with the recommendations of Nagasaki University. All experiments were approved by the Ethical Review Committee of the Institute of Tropical Medicine (NEKKEN), Nagasaki University, and were conducted according to the Animal Facility guidelines. The mice were divided into two experimental groups: the infected untreated group and the infected treated group. Each group contained 7–8 mice, and each mouse was percutaneously infected in the inguinal area with 50 *S*. *mansoni* cercariae. In the treated group, the mice were treated at 8 weeks post-infection with PZQ (Sigma, St. Louis, MO) at a dose of 500 mg/kg body weight for two consecutive days [33]. The drug was dissolved in distilled water, and 230 μl of the solution was administered orally by gavage. The mice were killed at the end of the experiment, and their livers and intestines were analyzed for the presence of eggs. Adult worms were also collected with perfusion of the portal vein to confirm infection and were counted to measure the worm burden. The effect of treatment was assessed by the improvement in symptoms such as anemia and the mobility of the mice and the reduction in ascites. One mouse was sacrificed 1 week after treatment (9 weeks post-infection), and seven mice were sacrificed at 18 weeks post-infection to confirm the absence of adult worms in the portal vein with perfusion, to confirm the efficacy of treatment. Five uninfected untreated mice were used as negative controls.

### Collection of sera from infected mice

Blood samples were collected biweekly from all mice in each group *via* the tail until 18 weeks post-infection. The blood samples were allowed to clot at room temperature for 2 h before centrifugation at 1,000 × g for 10 min at 4°C. The serum samples were collected into Eppendorf tubes and stored at −30°C until analysis. The sera collected from three uninfected mice were used as the negative controls.

### Preparation of crude SWAP and SEA and nine recombinant proteins

#### SWAP

Adult worms were harvested from mice infected with *S*. *mansoni* cercariae at 7 weeks post-infection by portal vein perfusion. The worms were washed three times with PBS, homogenized with a mechanical grinder, and centrifuged at 10,000 × g for 1 h at 4°C. The supernatant was collected and filtered through a 0.22 μm mesh. The protein concentration was measured with the Bradford method (Bio-Rad Laboratories, Hercules, CA) and the protein was then stored as aliquots at −30°C.

#### SEA

SEA was prepared as previously reported [34]. Briefly, purified eggs from the livers of infected ICR mice were resuspended in 5 ml of cold PBS at a concentration of 100,000 eggs/ml. The suspended eggs were sonicated for 15 min on ice (to prevent heating) until more than 95% were destroyed. The homogenized eggs were centrifuged at 2,000 × g for 20 min at 4°C. The supernatant was collected in a sterile tube and then ultracentrifuged at 100,000 × g for 1 h at 4°C. The supernatant was filtered, and the protein concentration was determined as for SWAP. SEA was as stored in aliquots at −30°C until use.

#### Recombinant proteins

Nine recombinant *S*. *mansoni* antigens, sm31, sm32, RP26, serpin, filamin, tropomyosin, GST, LAP1, and LAP2, were prepared as described elsewhere [21].

### Enzyme-linked immunosorbent assays (ELISAs) of serum antibodies

ELISAs were performed with different recombinant *S*. *mansoni* antigens as described elsewhere [21, 34], including sm31, sm32, RP26, serpin, filamin, tropomyosin, GST, LAP1, and LAP2. The SWAP and SEA crude antigens were also compared with the recombinant antigens of interest. For each recombinant antigen, Nunc MaxiSorp 96-well microtiter plates (Thermo Fisher Scientific, Waltham, MA) were coated overnight with 50 μl of antigen solution (5 μg/ml) per well (in PBS). For SWAP and SEA, the concentrations of the crude antigens were optimized to 200 ng/ml in a total volume of 50 μl. The plates were washed three times with PBS containing 0.05% Tween 20 (PBST) at room temperature and then blocked for 2 h with blocking buffer (1% bovine serum albumin [BSA; Sigma] in PBS) at room temperature. The plate wells were then washed with PBST, filled with mouse sera diluted 100 times with PBST containing 0.1% BSA, and incubated at room temperature for 2 h. After that plates had been washed as described above, they were incubated for 1 h with horseradish peroxidase (HRP)-conjugated goat anti-mouse IgG antibody (R&D Systems, Minneapolis, MN) diluted 1:1000 in PBST containing 0.1% BSA. The plates were washed, and the colorimetric substrate tetramethylbenzidine (BD Pharmingen, Allschwil, Switzerland) was added. The absorbance of the wells was measured at 450 nm with a Multiskan FC Microplate Photometer (Thermo Scientific). All serum samples were assessed in duplicate. The pre-infection sera taken from the same animals were used as negative control and also assessed for reactivity against each antigen to calculate the cut-off values.

Specific IgG1 and IgE antibodies were detected with sera from the infected mice (diluted 50-fold) collected biweekly, with an HRP-conjugated rat anti-mouse-IgE antibody (AbD Serotec, Bio-Rad) or HRP-conjugated rat anti-mouse-IgG1 antibody (Invitrogen) diluted 1:1000. The remaining ELISA steps were as described for the detection of total IgG.

### Parasitological examination

At the end of our experiment at 18 weeks post-infection, we killed all the mice in each group to check for the presence of adult worms, using portal vein perfusion, to assess the efficacy of the PZQ treatment.

### Statistical analysis

The statistical software GraphPad Prism 5.0 (GraphPad Software Inc., La Jolla, CA) and Excel were used for all statistical analyses and the generation of graphs. The ELISA cut-off values for the infected mice were estimated as the mean plus three standard deviations (mean + 3SD) of the optical density (OD) of the control group sera for each antigen. OD readings equal to or less than the cut-off value were considered negative, whereas those readings greater than the cut-off value were considered positive. ANOVA and a *t*-test were used for normally distributed samples, and the Mann–Whitney test was used for the other non-normally distributed samples. When comparing the differences between groups, p < 0.05 was considered statistically significant.

## Results

### Temporal dynamics of IgG raised against recombinant antigens during *S*. *mansoni* infection

**Fig 1:**
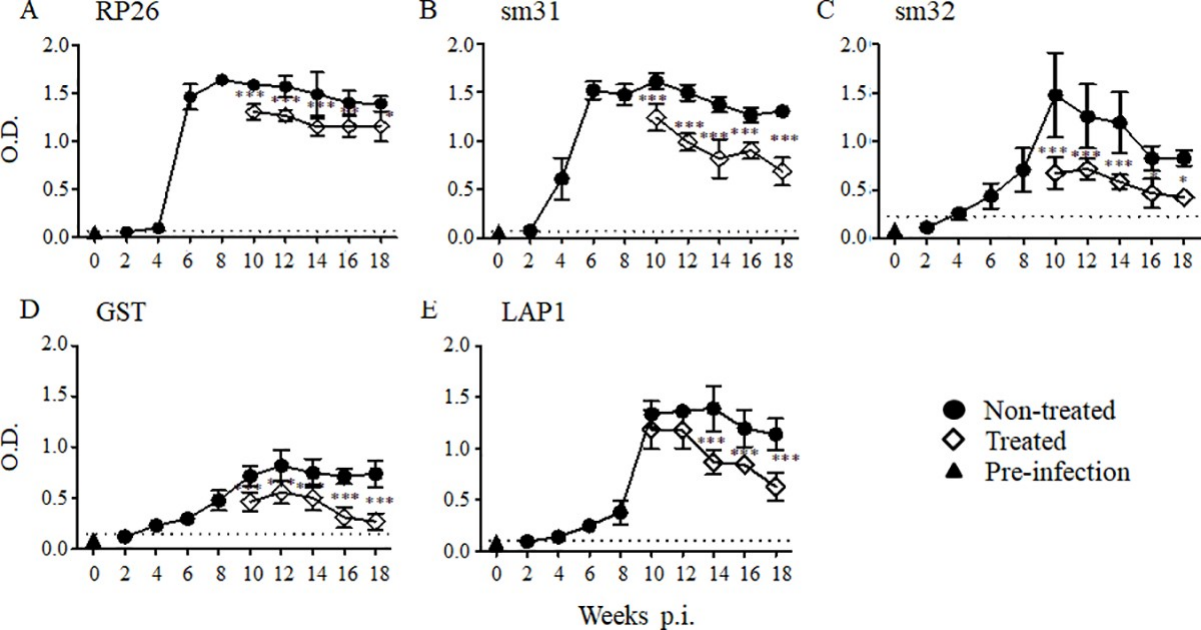
IgG responses to the *S*. *mansoni* antigens during *S*. *mansoni* infection and after the treatment. In each group, five mice were infected with 50 *S*. *mansoni* cercariae, and the sera were collected at each time point. A group of mice was orally treated twice with 500 mg/kg PZQ at 8 weeks post-infection. The experiment was repeated for 3 times and the representative data are presented. The means of the IgG level to the *S*. *mansoni* recombinant RP26 (A), sm31 (B), sm32 (C), GST (D), and LAP1 (E) are shown with the standard errors. The dashed line represents the cut-off value (mean + 3SD of the OD of the pre-infected group). The IgG levels of the treated group (open diamonds) are compared with those of the untreated group (closed circles) and with those of pre-infection samples taken at week 0 (closed triangles). *p < 0.05, **p < 0.01, and ***p < 0.001.

**Fig 2:**
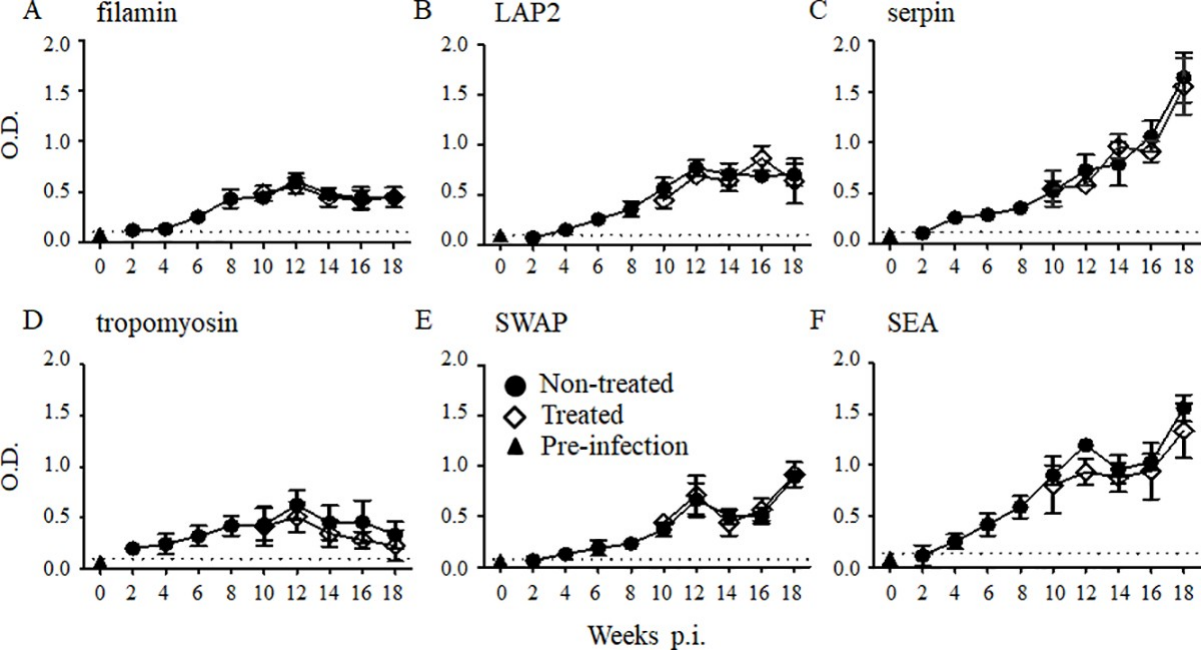
IgG responses to the *S*. *mansoni* antigens during *S*. *mansoni* infection and after the treatment. Five mice were infected with 50 *S*. *mansoni* cercariae, and the sera were collected from five mice at each time point. A group of mice was orally treated twice with 500 mg/kg PZQ at 8 weeks post-infection. The experiment was repeated for 3 times and the representative data are presented. The means of the IgG level to the *S*. *mansoni* recombinant filamin (A), LAP2 (B), serpin (C), tropomyosin (D), and crude SWAP (E) and SEA (F) are shown with the standard errors. The dashed line represents the cut-off value (mean + 3SD of the OD of the pre-infected group). Total IgG levels of the treated group (open diamonds) were compared with those of the untreated group (closed circles) and with those of pre-infection samples taken at week 0 (closed triangles).

Sera were collected from naïve and infected mice and measured for the total IgG raised against SWAP, SEA, or the nine recombinant *S*. *mansoni* antigens with ELISA. Most IgG raised against the recombinant antigens was first detectable in the sera at 4 weeks post-infection, as was the IgG raised against SWAP and SEA, whereas the IgG raised against tropomyosin was detected in the sera from 2 weeks post-infection, earlier than the other antigens **(Figs 1 and 2)**. The IgG raised against RP26 and sm31 rapidly and dramatically increased, peaking at 6 weeks post-infection **(Fig 1A and 1B)**. The IgG raised against sm32, GST, LAP1, filamin, LAP2, and tropomyosin peaked at 10–12 weeks post-infection, and then remained unchanged or decreased **(Figs 1C–1E, 2A, 2B and 2D)**. The IgG raised against serpin gradually and consistently increased during the infection, showing a similar pattern to the kinetics of IgG raised against both SWAP and SEA **(Fig 2C, 2E and 2F)**. The levels of IgG raised against RP26, sm31, sm32, LAP1, and serpin were higher than those raised against GST, filamin, LAP2, and tropomyosin, and similar to those of anti-SEA IgG **(Figs 1 and 2)**.

### Effects of PZQ treatment on the dynamics of IgG raised against recombinant antigens

To examine the effect of PZQ treatment on the kinetics of each IgG, mice infected with 50 *S*. *mansoni* cercariae were orally treated twice with 500 mg/kg PZQ at 8 weeks post-infection. The sera were collected every 2 weeks, and the IgG raised against each antigen was measured with ELISA. The IgG raised against RP26, sm31, sm32, and GST were significantly reduced at 2 weeks post-treatment **(Fig 1A–1D)**, whereas it took 6 weeks for the IgG raised against LAP1 to decrease significantly after the treatment relative to those in the untreated mice **(Fig 1E)**. For the remaining antigens, including SWAP and SEA, no significant reduction in IgG was observed after the treatment with PZQ compared with its level in the untreated group **(Fig 2)** at least until 10 weeks post-treatment. This result suggests that the antibody response to each antigen varies greatly during *S*. *mansoni* infection and after the treatment.

### Dynamics of IgG1 subtypes that react with RP26, sm31, and serpin during *S*. *mansoni* infection and after treatment

**Fig 3:**
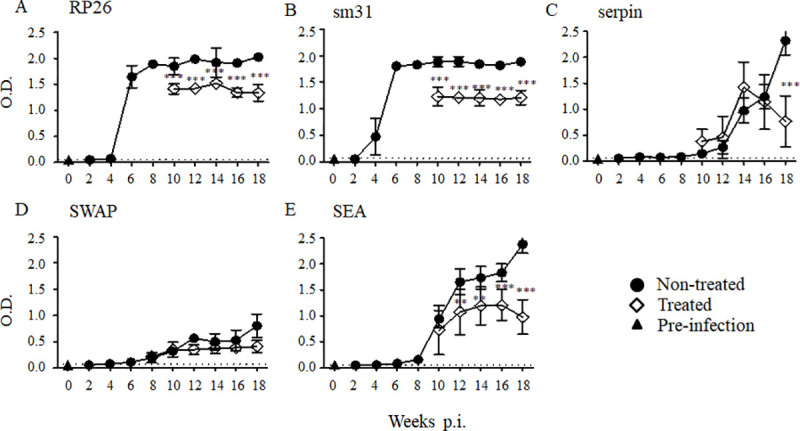
IgG1 responses to the *S*. *mansoni* antigens RP26, sm31, serpin, SEA and SWAP during *S*. *mansoni* infection and after the treatment. Five mice were infected with 50 *S*. *mansoni* cercariae, and the sera were collected from five mice at each time point. A group of mice was orally treated twice with 500 mg/kg PZQ at 8 weeks post-infection. The experiment was repeated for 3 times and the representative data are presented. The means of the IgG1 level to the *S*. *mansoni* recombinant RP26 (A), sm31 (B), serpin (C), and crude SWAP (D) and SEA (E) are shown with the standard errors. The dashed line represents the cut-off value (mean + 3SD of the OD of the pre-infected group). IgG1 levels of the treated group (open diamonds) were compared with those of the untreated group (closed circles) and with those of pre-infection samples taken at week 0 (closed triangles). **p < 0.01 and ***p < 0.001.

Previous immunological and epidemiological studies of *S*. *mansoni* infection have shown that a high level of IgG4 correlates with *S*. *mansoni* infection, and IgG4 is thought to be a useful biomarker of parasitic infection. In this study, IgG raised against RP26, sm31, and serpin were dramatically induced during the infection of mice, and their excellent diagnostic value has been demonstrated previously, with significant reactivity in patient plasma [21, 33]. Because murine IgG1 is equivalent to human IgG4, we analyzed the IgG1 raised against RP26, sm31, and serpin during infection and after the treatment. The IgG1 raised against crude SWAP and SEA were induced at 4 weeks post-infection compared with the levels in uninfected mice. The IgG1 levels to both SWAP and SEA gradually increased during the infection **(Fig 3D and 3E)**. The IgG1 raised against RP26 and sm31 were detected in the sera of mice at 4 weeks post-infection **(Fig 3A–3C).** The IgG1 that reacted to RP26 and sm31 increased dramatically by week 6 post-infection and plateaued, whereas those raised against serpin increased gradually during the whole period of observation, similar to the IgG that reacted to it. The IgG1 raised against RP26 and sm31 declined significantly 2 weeks after the treatment **(Fig 3A–3C)**, whereas the IgG1 to serpin declined significantly by 10 weeks post-treatment. The IgG1 raised against crude SEA declined by 4 weeks post-treatment, too **(Fig 3E)**. The kinetics of the IgG1 levels to the recombinant antigens during infection and after the treatment tended to be similar to those of IgG for all antigens, indicating that the detection of IgG1 and IgG4 in humans may be an alternative to the detection of total IgG.

### Dynamics of IgE raised against sm31, RP26, and serpin during infection and after treatment

**Fig 4:**
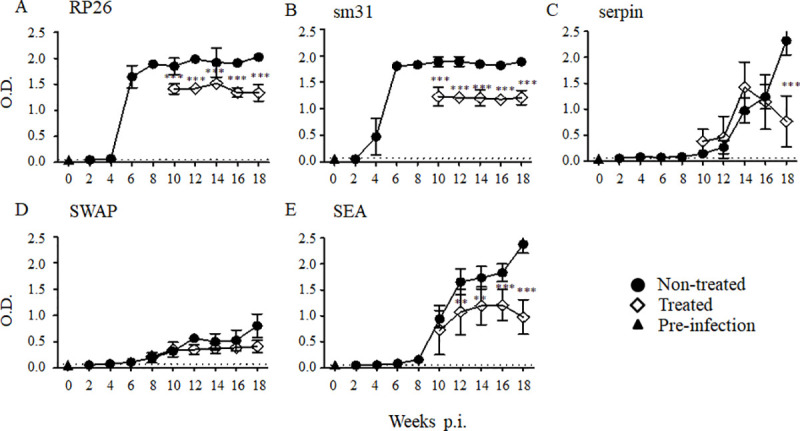
IgE responses to the *S*. *mansoni* antigens RP26, sm31, serpin, SEA and SWAP during *S*. *mansoni* infection and after the treatment. Five mice were infected with 50 *S*. *mansoni* cercariae, and the sera were collected from five mice at each time point. A group of mice was orally treated twice with 500 mg/kg PZQ at 8 weeks post-infection. The experiment was repeated for 3 times. The means of the IgE level to the *S*. *mansoni* recombinant RP26 (A), sm31 (B), serpin (C), and crude SWAP (D) and SEA (E) are shown with the standard errors. The dashed line represents the cut-off value (mean + 3SD of the OD of the pre-infected group). IgE levels of the treated group (open diamonds) were compared with those of the untreated group (closed circles) and with those of pre-infection samples taken at week 0 (closed triangles). **p < 0.01 and ***p < 0.001.

Because IgE is known to be associated with IgG4, we next examined the reaction of IgE with recombinant proteins RP26, sm31, and serpin. The results showed that the IgE raised against sm31 and serpin were induced by 2 weeks post-infection, before the onset of egg production, which was similar to the induction of IgE to SWAP **(Fig 4B–4D)**. The IgE raised against RP26 and sm31 remained high, even after the treatment, whereas the IgE raised against serpin declined significantly by 6 weeks post-treatment **(Fig 4A–4C)**. There was no change in the IgE bound to SWAP or SEA after the treatment (**Fig 4D and 4E)**. The IgE raised against each antigen remained elevated after the treatment with PZQ, except the IgE raised against serpin, which declined significantly after the administration of PZQ.

## Discussion

In this study, we examined the dynamics of the serological responses to the nine recombinant *S*. *mansoni* antigens during *S*. *mansoni* infection, before and after the treatment with PZQ, and evaluated their potential as sensitive markers to monitor schistosome transmission in low-endemic settings, especially after the MDA with PZQ.

The IgG to all the tested antigens were detected within 4 weeks after the infection. The IgG raised against some of the recombinant antigens were robustly induced in the early phase of infection and maintained at high levels, whereas reportedly a longer infection period is required to induce IgG responses to most *S*. *mansoni* antigens in an endemic community [35]. The IgG to SEA and SWAP were maintained at high levels and were not affected by the treatment with PZQ, which is consistent with the observation that they persist for years after the clearance of the adult worms [35]. Among the nine selected candidate antigens, total IgG to sm31, sm32, RP26, LAP1, and GST significantly declined after the treatment with PZQ, indicating that they can be promising candidates in the study to further evaluate antigens for monitoring the transmission of schistosomiasis after MDA, facilitating the control and elimination of the disease [36, 37]. However, the levels of IgG were still higher than the baseline levels at only several weeks after the treatment. This property should be prospectively examined in human sera before and after the treatment, and it will be useful to clarify how many months are required for the IgG directed against each antigen to decline below the baseline level after the treatment with PZQ. These results suggest that the selected schistosome antigens in our study have significant potential for the monitoring and surveillance of schistosomiasis.

In this study, we analyzed the kinetics of IgG1, corresponding to IgG4 in human, and IgE directed against selected recombinant antigens. There were drastic increases in the IgG1 and IgE responses to sm31, RP26, and serpin. Interestingly, although the levels of IgG1 raised against RP26 and sm31 declined soon after the PZQ treatment to some extent with significance, IgG1 to serpin significantly declined at 10 weeks after the treatment. The kinetics of the IgG1 response to SEA in this study were similar to those reported by Matoso et al. [38]. Their study was conducted with human sera and suggested that anti-SEA IgG4 reactivity can be used as a biomarker for the serological monitoring of *S*. *mansoni* infection in endemic areas [38]. Therefore, it would be important to examine the IgG4 responses to sm31, RP26, and serpin using sera collected before and after the treatment with PZQ.

Helminthic infections, including *S*. *mansoni*, are known to induce IgE responses [39]. A study about antibody production during lymphatic filariasis suggested that the induction of IgG4 subtype and IgE antibodies was coordinately regulated [40] and that the immunoglobulins were often raised against the same antigens [41]. Previous studies have often analyzed the crude antigens of a parasite. As those preparations contain multiple allergens that are exposed to the host immune response under different conditions, it is difficult to determine which ones are responsible for the dual production of IgE and IgG4. By using recombinant proteins in this study, we were able to confirm the specific dual production of IgG1 and IgE raised against recombinant antigens during schistosome infection. In the present study, most of the antigens induced a similar pattern for IgG4 and IgE production, corroborating the report by Hussain et al. [40]. It is noteworthy that the specific IgG1 and IgE raised against the serpin antigen displayed similar patterns both before and after the treatment, insofar as they decreased simultaneously after the treatment. This combination of specific IgG1 and IgE raised against serpin could be used to monitor *S*. *mansoni* reinfection after MDA.

In summary, in this study, we have shown that the recombinant RP26, sm31, sm32, GST, LAP1, and serpin antigens can be good candidates in selecting potential markers in the study using human sera before and after the treatment for guiding programs to eliminate schistosomiasis.
