# Correction: Dynamics of serological responses to defined recombinant proteins during *Schistosoma mansoni* infection in mice before and after the treatment with praziquantel

**DOI:** 10.1371/journal.pntd.0010309

**Published:** 2022-03-22

**Authors:** Eman Sayed Mohammed, Risa Nakamura, Yombo DJ Kalenda, Sharmina Deloer, Taeko Moriyasu, Mio Tanaka, Yoshito Fujii, Satoshi Kaneko, Kenji Hirayama, Ahmed I. Ibrahim, Mahmoud A. El-Seify, Asmaa M. Metwally, Shinjiro Hamano

Fig 4 appears as a duplicate of Fig 3. Please view the correct Fig 4 below.

**Fig 4:**
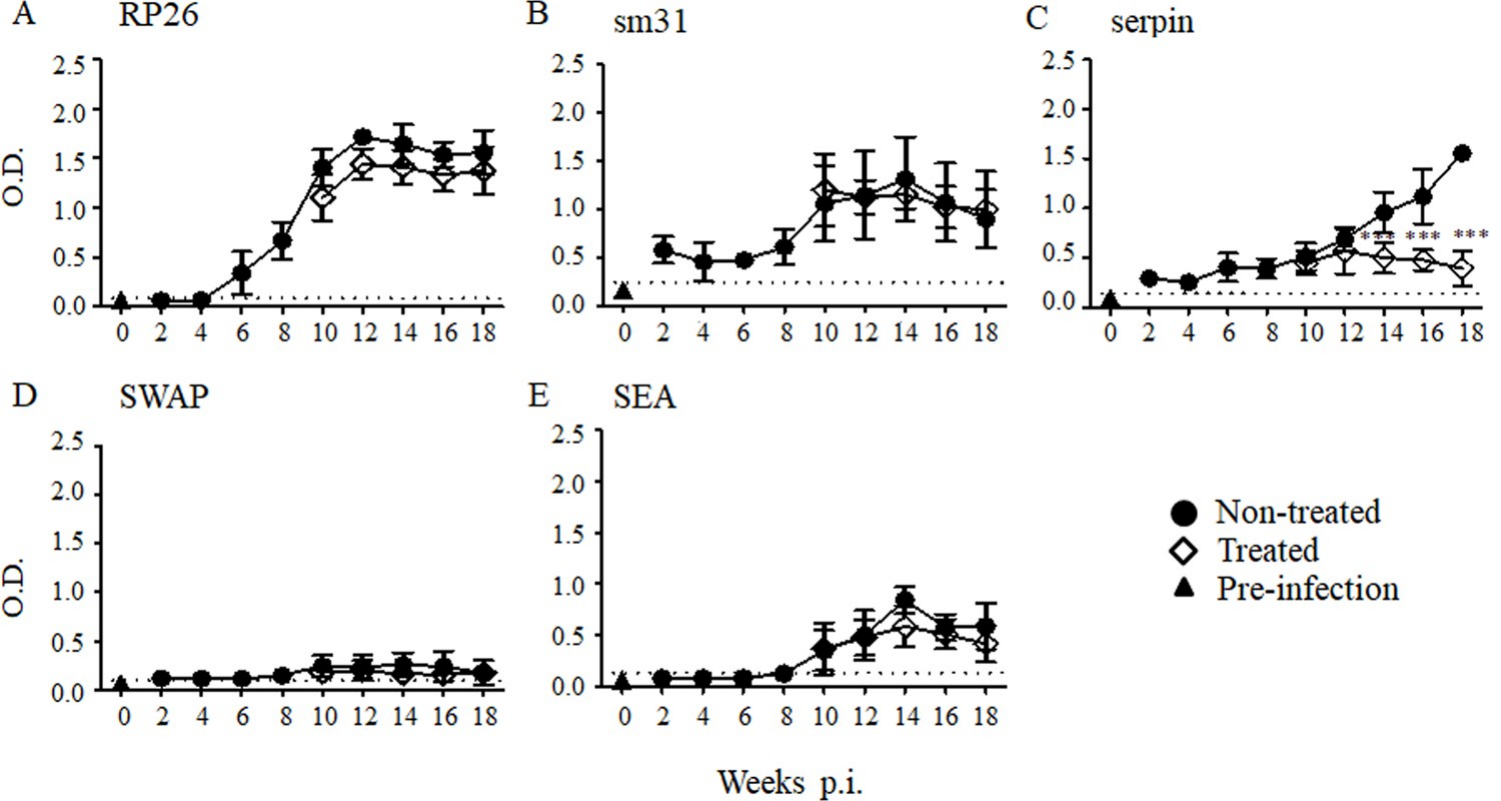
IgE responses to the *S*. *mansoni* antigens RP26, sm31, serpin, SEA and SWAP during *S*. *mansoni* infection and after the treatment. Five mice were infected with 50 *S*. *mansoni* cercariae, and the sera were collected from five mice at each time point. A group of mice was orally treated twice with 500 mg/kg PZQ at 8 weeks post-infection. The experiment was repeated for 3 times. The means of the IgE level to the *S*. *mansoni* recombinant RP26 (A), sm31 (B), serpin (C), and crude SWAP (D) and SEA (E) are shown with the standard errors. The dashed line represents the cut-off value (mean + 3SD of the OD of the pre-infected group). IgE levels of the treated group (open diamonds) were compared with those of the untreated group (closed circles) and with those of pre-infection samples taken at week 0 (closed triangles). **p < 0.01 and ***p < 0.001.
